# A formative research to explore the programmatic approach of vaccinating the Rohingya refugees and host communities against COVID-19 infection in Bangladesh

**DOI:** 10.1186/s12913-023-09945-z

**Published:** 2023-08-31

**Authors:** Anika Tasneem Chowdhury, Satyajit Kundu, Zeeba Zahra Sultana, Heba Hesham Ali Hijazi, Ahmed Hossain

**Affiliations:** 1 Department of Public Health, North South University, Dhaka, 1229 Bangladesh; 2 Global Health Institute, North South University, Dhaka, 1229 Bangladesh; 3 Department of Public Health and Primary Care, University of Cambridge, Cambridge, UK; 4 College of Health Sciences, University of Sharjah, Sharjah, 27273 United Arab Emirates; 5 Faculty of Medicine, Jordan University of Science and Technology, Irbid, 22110 Jordan

**Keywords:** Rohingya, COVID-19, Vaccine, Bangladesh, Formative research

## Abstract

**Background:**

The vaccination of the Rohingya refugees and host communities against COVID-19 in Cox’s Bazar started in August 2021. Government authorities and Non-Government Organisation partners implemented a project around the initial period of vaccination to improve awareness and access to target beneficiaries. We conducted formative research to understand the programmatic approach of this project and identify potential challenges and community perceptions regarding immunisation against COVID-19.

**Methodology:**

This was formative research in which we used a qualitative method of data collection. Purposively chosen 12 key-informant interviews and conveniently chosen 20 in-depth interviews were conducted using semi-structured interview guidelines from August to September 2022 in the Rohingya camp and host communities of Cox’s Bazar District, Bangladesh. Ethical approval was obtained from the North South University Institutional Review Board, and written informed consent was obtained from all the participants. We used a thematic analysis approach to analyse the data.

**Results:**

The project neither provided any promotional or tailored messages regarding the COVID-19 vaccine nor conducted any vaccine hesitancy surveys before implementing the project. The project did not provide any storage facilities for the vaccines’ cold chain management but provided transport support to carry the vaccines from the district to the sub-district level. Community leaders were included in the decision-making process during local-level planning of the vaccination programme. The project supported the reporting of any adverse effects following immunisation from community members to the government health authorities. Vaccine hesitancy among participants was high in the early stages, but mass campaigns and vaccination of frontline health workers increased their acceptance. The major challenges reported by the informants were low budget and lower salaries of field staff, stacking of the registration process at the beginning, reluctance of participants, inadequate transportation and manpower, and inadequate baby feeding corners at vaccination centers.

**Conclusion:**

The findings from our study will help policymakers from the Government, the UN, and other humanitarian agencies to adapt and better address the issue of vaccine acceptance and strengthen the vaccination programme.

**Supplementary Information:**

The online version contains supplementary material available at 10.1186/s12913-023-09945-z.

## Background

While 2020 was marked by the fear of COVID-19 infection, 2021 would be remembered as the year of finding a solution to this global pandemic, the COVID-19 vaccine [1, 2]. Bangladesh started a target-specific COVID-19 vaccination programme on 27 January 2021, and mass vaccination from 7 to 2021 [3, 4]. Cox’s Bazar is one of the poorest districts in Bangladesh and is considered to be at the eighth highest composite risk of exposure to COVID-19 infection [5–7]. The Teknaf and Ukhiya sub-districts of Cox’s Bazar district have a population of approximately 310,000 and 240,000, respectively [8]. In addition, Cox’s Bazar hosts about 919,000 Rohingya refugees at Teknaf and Ukhia refugee camps, making these places among the most densely populated camps in the world and more vulnerable to the transmission of COVID-19 infection [9]. According to the World Health Organization (WHO) situation report, published on 26 September 2021, Bangladesh confirmed more than 1,700,000 COVID-19 cases, among which 17,311 were identified in Cox’s Bazar District, including 3,084 Rohingya refugees [8]. The Government of Bangladesh planned to start vaccination against COVID-19 in the Rohingya camps in Cox’s Bazar from March 2021, but due to the disruption of shipment from India, the vaccination programme was delayed until August 2021 [10, 11]. WHO, together with other health sector partners and donors, strongly advocated for the delivery of vaccines to Bangladesh and managed to include Bangladesh in the top four countries to prioritise vaccine delivery under the COVAX programme [6]. In line with the Joint Response Plan (JRP) and coordination with the health sector, Non-Government Organisations (NGO) aimed to complement the Government’s COVID-19 vaccination rollout for vulnerable refugee camps and host communities from September to October 2021 [7]. Just before the planned project started, an assessment by WHO aimed at understanding community preparedness for COVID-19 vaccination showed that 76% of Rohingya did not know that the COVID-19 vaccine was available in Bangladesh, nearly 83% did not realise that vaccination could start soon, and 98% did not understand the leaflet circulated on COVID-19 vaccination [8]. Previous experience showed that the information gap, lack of mass awareness, sensitisation, and fewer community mobilisation hampered Bangladesh’s first phase of the vaccination programme [8].

A study conducted in Germany by Antonia Bendau et al. showed that health-related fear and anxiety were significantly correlated with vaccine acceptance among the participants (p-value < 0.001) [12]. Another study conducted in Portugal revealed that confidence in the effectiveness of the vaccine greatly influenced COVID-19 vaccine uptake [13]. Therefore, it was pertinent to understand the perception of the Rohingyas and host community members if the vaccination programme was to be successful. There were also gaps in the plan to reach the most vulnerable people, especially people with disabilities (PWDs), the elderly above 60 years, and pregnant and lactating women (PLWs) in this minority group. These vulnerable groups are at greater risk of having severe symptoms of COVID-19 and face challenges in accessing health services.

A rapid behavioural assessment of the Rohingya community in Bangladesh was conducted by Mohamed F. Jalloh et al. that explored the perception and acceptance of other vaccines of the refugees before COVID-19 [14]. According to the study findings, advocacy through the community leaders, messages disseminated from the mosque, community meetings, counselling by the community health workers during domiciliary visits were the facilitators of vaccine uptake, whereas safety concerns, and cultural barriers were cited as the chief barriers against the programme [14]. Research briefs, commentaries, and newspaper articles highlighted several key facts on the COVID-19 vaccination programme and community perception among Rohingya refugees [15–17]. However, evaluation of the programme and community acceptance of the vaccine have not been explored qualitatively.

An 11-month project in the Teknaf and Ukhiya sub-districts of Cox’s Bazar district was implemented from 1 to 2021, to 30 June 2022, and the project stakeholders were the government authorities and NGO partners. The four pillars of the project were - vaccine administration, risk communication, community engagement, and surveillance of adverse effects. Vaccine uptake was low in the refugee community at the beginning, which created the need for a formative research to refine the vaccination campaign in an iterative process [18]. Therefore, we conducted formative research with an aim to understand vaccine acceptance in the study communities, explore the programmatic approach of the vaccination project, and identify the potential gaps in the system. The findings of this study set up a baseline upon which the Government, the UN, and other humanitarian agencies were able to adapt and develop a better plan to achieve the target of the vaccination programme.

## Methodology

The consolidated criteria for reporting qualitative research (COREAQ) were used to report the findings of this study.

### Study design and study settings

This formative research was conducted in Cox’s Bazar District of Bangladesh from August 2022 to September 2022. The study regions were divided into two communities: Rohingya and host communities. Informants from the Rohingya community were selected from the Ukhiya sub-district (21.2447^0^ N, 92.1339^0^ E) and the Teknaf sub-districts (20.8578^0^ N, 92.2967^0^ E), while informants from the host community were selected from the Ramu (21.4324^0^ N, 92.1008^0^ E) and Chakaria (21.7619^0^ N, 92.0776^0^ E) sub-districts.

### Sampling technique and sample size

The main study included 546 households from the Rohingya community and 553 households from the host community, using a two-stage cluster sampling technique. From the selected households, we interviewed informants until data saturation occurred. In this process, 20 in-depth interviews and 12 key-informant interviews were conducted. The 10 informants from the Rohingya community and 10 informants from the host community were interviewed at the convenience of the data collectors based on their interest in participating and the availability of the informants at the time of visit to their households. The 12 key informants were selected purposively so that programmatic initiatives, community approaches, and gaps in the system could be properly captured.

### Data collection

Semi-structured interview guidelines were used for key-informant and in-depth interviews. The guidelines were developed in Bangla for the convenience of both the interviewers and interviewees and contained open-ended pre-defined questions in a logical order to ensure that all the research questions were addressed without hampering the natural flow of conversation. We developed a-priori code before the interviews. During the data collection period, the interviewers and project researchers had intermittent discussions on the collected information and added all the emerging codes from the ongoing interviews or made necessary modifications to capture the complete flow of data. The codebook is added with Supplementary file – 1. Five trained and experienced qualitative researchers conducted the interviews. The informants were contacted before the interviews, and the place of interviews was selected based on their convenience. In-depth interviews were conducted at the informants’ households, and key-informant interviews were conducted at their workplaces. We audio-recorded all the interviews and did verbatim transcription. The key-informant interviews took 20 min and the in-depth interviews took 45 min on average.

### Data analysis

We used a thematic analysis approach to outline, describe, and report the key patterns within and across the theme-wise responses. One matrix-based coding frame was developed, for both the KII and IDI transcripts. Themes and sub-themes were prepared after repeated reading of the transcripts, and the codebook was finalised for analysis.

## Results

To understand the perceptions of the host and Rohingya community regarding vaccination uptake, we conducted 20 in-depth interviews. The distributions are listed in Table 1.

**Table 1:** Distribution of the informants of the in-depth interview

In-depth interview	Host community (n)	Rohingya community (n)
**Age group**		
** 20–29 years**	6	1
** 30–39 years**	2	4
** 40–49 years**	0	2
** 50–59 years**	0	1
** > 60 years**	2	2
**Sex**		
** Male**	7	5
** Female**	3	5
**Vulnerability**		
** Pregnancy**	2	1
** Lactating mother**	1	1
** Disable**	1	1

To gather knowledge about the health system weaknesses that plagued the COVID-19 response, twelve key informants were selected equally from both communities that were considered capable of providing the needed information. The spread has been provided in Table 2.

**Table 2:** Distribution of the informants of the key informant interview

Identity	Number (N = 12)	Gender distribution (M:F)	Roles
**Refugee Community**	4	3:1	Boat man (1), Teacher (1), Religious leader (1), Common people (1)
**Host Community**	4	2:2	Religious leader (1), Community leader (1), Community people (2)
**NGO Professionals**	4	2:2	Administrative personnel (1), Involved in vaccine distribution (3)

**Fig. 1:**
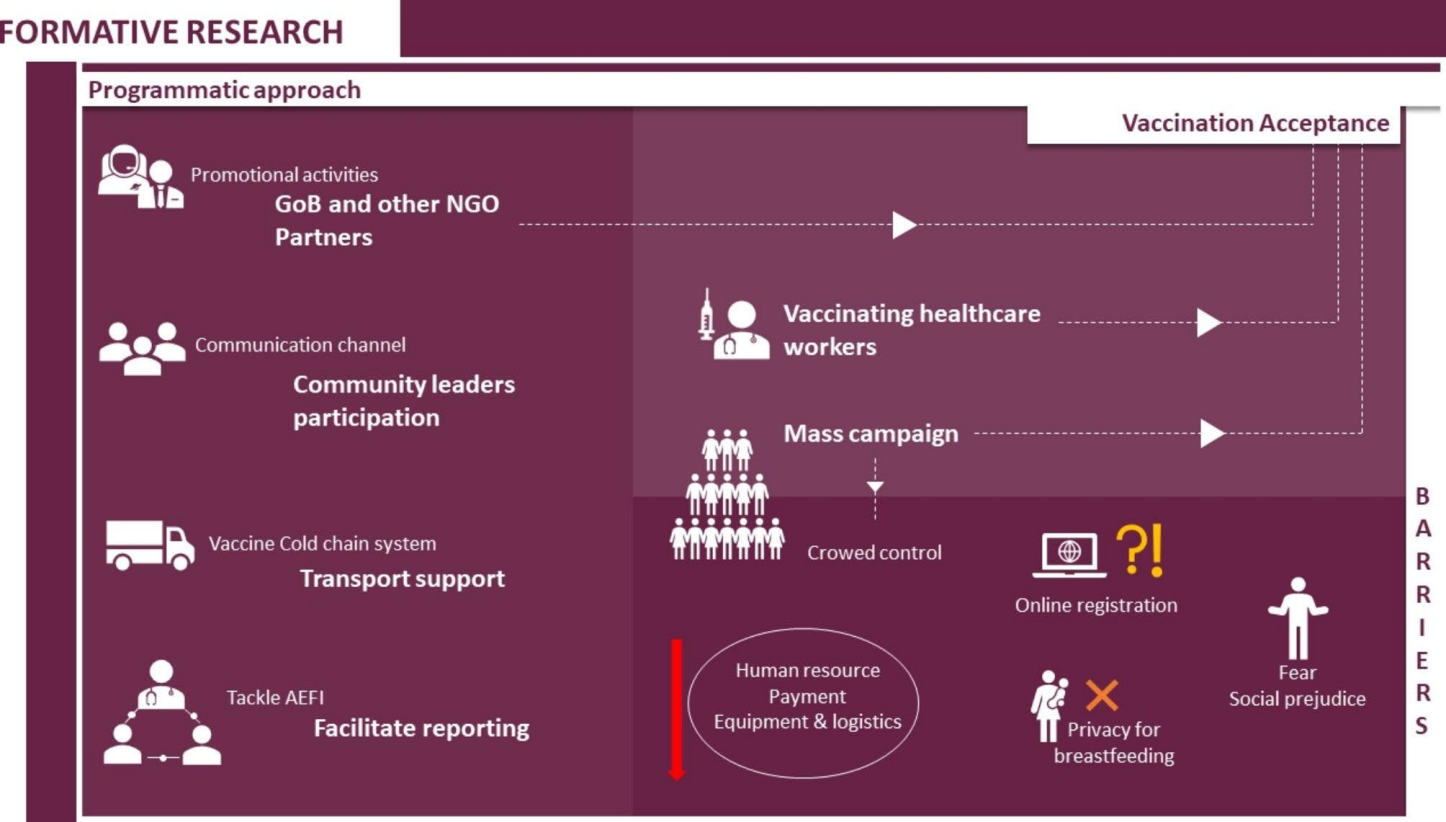
Thematic areas of the study

The programmatic approach, vaccination acceptance, and barriers to the programmes were the main themes identified in this study. Themes are shown in Fig. 1.

### Vaccination acceptance among the informants

According to the key informants, both the host and Rohingya populations were initially hesitant about taking the vaccine, especially the pregnant and elderly believed that they did not meet the inclusion criteria of vaccination. In particular, the Rohingya community was more conservative about taking the COVID-19 vaccine than the host community. The deaths of people due to COVID-19 infection following a single dose of the vaccine aggravated scepticism. However, this sceptical attitude disappeared after the mass vaccination programme. One of the key informants from the Rohingya Camp mentioned:

*People died from COVID after receiving their first dose during that period; people asked us, “Why should we take the vaccine if it kills people*?”

Initially, the vaccine acceptance rate was low due to misinformation and fear about the vaccine’s possible side-effects. Vaccine phobia was higher in the Rohingya community than in the host community. However, their hesitancy was gradually erased after circulating the tailored messages, and when community members saw doctors and frontline healthcare workers taking the vaccine. The influence and awareness campaigns by the government played a crucial role in enhancing vaccine acceptance among the community.

Post-vaccination side-effects did not alter the acceptance of the COVID-19 vaccine among study informants after passing the initial phase. Among the host and Rohingya communities, the most widely reported symptoms were minor degree of fever, headache, body ache, stomach ache, swelling, and pain at the vaccination site. Most of them were reported to healthcare providers. They took painkillers for their symptoms, prescribed by their doctors or self-medication.

My family and I never got any serious post-vaccination symptoms.

(Male, host community)

My family and I never got any serious post-vaccination symptoms, but I got a fever and pain in my body, we reported it to the service providers, and they asked us to take paracetamol.

(Female, Rohingya community)

### Programmatic approach

#### Promotional activities: vaccine hesitancy survey and tailored messages

The vaccination programme started on February 7, 2021, and the project started no later than a few months later. Most of the key informants agreed that the project did not support any vaccine hesitancy survey before its implemention. The World Health Organization conducted a vaccination needs assessment survey, and the project was designed based on this study. Therefore, they did not require additional data. A senior officer from the stakeholder added:

We did not conduct any vaccine hesitancy surveys for COVID-19 vaccination. However, the community people were influenced by the Government of Bangladesh and NGO’s field-level staff to take the vaccine through mass media.

The key informants from the government authority reported that vaccine promotional activities were running before starting the project and continued during project implementation. The Government of Bangladesh broadcasted tailored messages on the COVID-19 vaccine in the locality, along with local non-government organisations. Promotional activities included pamphlet distribution, miking, home visits, and other outreach activities. Additionally, when the community was embedded with different misinformation and taboos, the project staff made sure to engage the community and religious leaders to tackle the problem. An officer from government administration mentioned,

Yes, the NGO and the organisations of the Government of Bangladesh broadcasted tailored messages regarding COVID-19 vaccination periodically.

All the informants of the host and Rohingya communities unanimously agreed that they received promotional messages on COVID-19 vaccination via miking, television advertisements, newspapers, relatives, neighbors, community leaders, different NGOs, doctors, and volunteers.

Yes, we received promotional or tailored messages regarding COVID-19 and its vaccine via TV, newspaper, local member and miking.

(Male, host community)

We received different tailored messages to take the COVID-19 vaccine through miking, doctors and volunteers.

(Male, Rohingya community)

#### Inclusion of the community leaders in the decision-making process

Community leaders were directly involved in the decision-making process and acted as channels between community members and policymakers. At the beginning, the pregnant women, and lactating mothers were excluded from the vaccination programme, but were included soon following the orders of the government. Additionally, the pregnant women and the elderly received proper vaccination coverage in the Rohingya community due to the advocacy of community leaders and the advantage of having a separate transport facility. A Community Healthcare Manager reported,

These groups were taken into consideration during the decision-making process. Pregnant or breastfeeding women, people with disabilities, and those over 60 years of age were all adequately immunised in the Rohingya community with transportation assistance. However, we were unable to adequately cover them in the host community.

#### Cold chain and quality of the vaccines

The project did not provide any storage facilities for cold chain management of vaccines. However, the project supported the transport facilities of vaccines from district storage to sub-district storage systems using a specialised vehicle with a temperature control system. The storage systems for the vaccines were under the administration of the Government of Bangladesh at the sub-district level. The two sub-districts of the project area had a specialised cold storage system to store vaccines. The upazila health administrator was responsible for maintaining the proper temperature and quality of the vaccine before implementation. The safety and proper temperature of the vaccines were maintained and inspected by the Upazila Health and Family Planning Officer (UH&FPO) of each Upazila. There was good cooperation and understanding between the NGO and the respective government wing of Bangladesh to handle the cold chain of vaccines. In addition, there was no scarcity of the availability of the vaccine during the project because at the time of the implementation, the government was supplying adequate vaccines as a part of the mass vaccination program. As a result, there was no shortage of vaccines.

It was quite helpful for them to maintain the cold chain of the COVID-19 vaccine since the project supported transportation facilities with a specialised vehicle. Besides, we were always involved in inspecting the safety and appropriate temperature of the cold storage of the vaccine.

(UH&FPO)

#### Preparatory mechanism to tackle unintended adverse effects of the vaccine

The NGO was not responsible for managing the adverse effects or other severe consequences of vaccination; rather, it was the government’s responsibility. The healthcare workers of the public facilities were trained on the management of side effects, and mobile numbers of doctors were circulated to the community leaders and community members for continuous service coverage. Vaccine providers counselled the recipient on potential side-effects and sought care as soon as symptoms developed. Additionally, there was a reserved bed in government healthcare facilities at the Upazila level as a precautionary measure. However, the project supported subsequent reporting from the community to government health authorities. An expert from an NGO stated,

We were not responsible for managing the adverse condition of vaccinated people, while we are always ready to support the Government by providing vehicle support for transportation and referral of adverse cases. However, to our knowledge, there have been no widespread reports on the significant side effects of vaccination. After the immunization, we monitored them and provided them counselling on possible side effects.

### Barriers to the programme

The initial challenge was to motivate the community to accept this vaccine. Inadequate human resources, equipment, and logistics, a need for incentives to bring the poorer group, and lack of proper communication were reported by the key informants that stood between the success of vaccine coverage. The project provided only food and transportation costs for healthcare assistants and volunteers, and there was no remuneration for them. The informants from both the host and Rohingya communities also complained about the difficult registration process.

The vaccine storage facility on the remote location fridge and the vaccine center’s air conditioning was required.

(Community Outreach Officer)

Because there was no budget for this, it was difficult to reach out to remote areas for follow-up.

(UH&FPO)

We faced some obstacles to getting the vaccine, such as waiting to follow the queue and registration process.

(Male, host community)

Additionally, a conservative society, social prejudice, and illiteracy aggravated problems in the host community.

We faced some obstacles to getting the vaccine, such as vaccination phobia. Initially, we were scared about it, but now it’s easy to get the vaccine.

(Male, host community)

This issue was resolved through awareness programme. The difficulty in crowd control was then the new challenge. The informants also found it difficult to get the vaccine and waiting in a long queue was bothersome to them. Another barrier was the lack of privacy to breastfeed their infants while waiting for the vaccine.

We faced obstacles to getting the vaccine, such as waiting to follow the queue. Initially, we thought it was easy to get vaccines, but now it is tough because of the long queue.

(Male, Rohingya community)

*We faced some obstacles to getting the vaccine, such as waiting to follow the* queue and not feeding the baby while waiting in line.

(Female, Rohingya community)

Transport was another challenge that was partially resolved in the Rohingya camp, which received the government support in this regard. Community leaders’ engagement also proved to be an effective strategy to overcome these challenges.

The government collaborates with us to overcome barriers or challenges. The NGO and the Government collaborated synergistically.

(Senior Health coordinator)

To reach people in remote areas, we included community leaders, chairmen, UP members, teachers, and religious leaders.

(Community Outreach Officer)

#### Opinion of the informants to overcome the barriers

The key informants recommended conducting a need assessment before starting the programme, making proper plans, and ensuring adequate human resources, equipment, and logistics to enhance the effectiveness of the intervention. The project beneficiaries also mentioned that adequate transport facilities and monetary support, especially for pregnant women and people with disabilities, would be much more helpful for them to get the vaccine because these vulnerable groups were sometimes unable to reach the vaccine centers.

To reach all the people from the host community, it was required to increase the project duration, manpower, remote area vaccination camp, transportation assistance for people in remote areas.

(Community Outreach Officer)

It is tough to get vaccines; it will be easy if we get some money and transport facility for vaccination.

(Male, Rohingya community)

## Discussion

The objective of our study was to explore the vaccination programme, identify the barriers, and understand community acceptance of the COVID-19 vaccine in the Rohingya and host communities of Bangladesh. Vaccine hesitancy among participants was high in the early stages, when vaccine programmes were about to be implemented. This behaviour was also initially observed among residents of other parts of the world, such as Kuwait, Jordan, Italy, Russia, Poland, the United States, and France [19]. The unavailability of vaccines in the first phase, breakthrough infection following vaccination, and deaths due to the infection itself, even after vaccination, were the possible causes of initial hesitancy [3, 20–22]. However, suspicion and ambivalence towards the COVID-19 vaccine among the Rohingya and host communities diminished over time. William Douglas Evans and Jeff French emphasised that confidence on the supply side, promotional activities, and service marketing are the key to increasing the acceptance of the COVID-19 vaccine among the general population [23]. Although the project did not provide a cold chain system, it facilitated in the transportation of vaccines from the district to the sub-district level, and there was no shortage of vaccine supply once it started in the project area. As a result, supply-side confidence could be established in vaccination programme. Vaccine hesitancy surveys and promotional activities were not directly exercised in this project, but most beneficiary informants participating in the qualitative study reported receiving promotional and tailored messages regarding the COVID-19 vaccine from NGO. It seems that the promotional activities by the Government of Bangladesh and other NGOs were widespread, and the project beneficiaries thought that they were part of the project implemented by the NGO and served the purpose of promotional activities. Additionally, vaccinating healthcare providers at the initial stage and mass campaigns helped in service marketing.

Although fear of post-vaccination side-effects initially reduced vaccine acceptance among the study informants, the mild nature of the symptoms overcame this obstacle after passing the initial phase. Regarding post-vaccination symptoms, participants reported very mild symptoms, and not everyone reported their symptoms to the authorities. The findings are similar to the study findings of Abanoub Riad et al., who reported absent to mild side-effects following COVID-19 vaccination among the study informants [24]. The staff who administered the vaccine counselled the patients to report any symptoms following vaccination and to seek proper care. The majority of the vaccine recipients were compliant and reported to healthcare providers. However, some of the informants self-medicated due to the mild nature of the symptoms. This behaviour could lead to, although rare, major health problems, such as anaphylatic reaction [25]. Rigorous counselling and health education are needed to create awareness among all the community people regarding the adverse effects of immunisation and steps that should be followed if any such incident occurs. A proper flow of correct knowledge on the possible side-effects and steps of management will reduce vaccine-centred fear and increase vaccine uptake as evidenced by a study conducted on refugees in Australia [26]. Design and distribution of proper behaviour change communication materials and counseling by professional healthcare workers can exert a beneficial impact in promoting vaccination as well as creating awareness about the possible side effects [26, 27].

The project engaged community leaders, provided transport facilities from the community to the vaccine centre, helped in vaccine transport, and tracked community cases of vaccine recipients for the development of any adverse effects. Similar approaches have also acted as facilitators of COVID-19 vaccine uptake among refugees and minority groups across different parts of the world [26, 28, 29]. Community leaders’ involvement in the decision-making process during the local-level planning of the vaccination programme is noteworthy. A study conducted in Australia found that community engagement, especially leaders’ involvement, encouraged vaccine uptake among refugees [26]. According to Arnstein, community participation can be achieved at multiple levels, depicted as a “ladder” [30]. The levels are degrees of citizen power including citizen control, delegated power, and partnership; degrees of tokenism including placation, consultation, and informing; and non-participation, including therapy and manipulation [30]. This project engaged the community at degrees of citizen power and tokenism that helped the vaccination programme to run smoothly and increased vaccination acceptance among the community people as community leaders acted as a bridge between the common people and policymakers.

The major challenges reported by the informants were low budget and lower salaries of field staff, stacking of the registration process at the beginning, reluctance of participants, inadequate transportation and human resources, and inadequate baby feeding corners at vaccination centers. The barriers identified in this study are similar to those identified in studies conducted among refugee populations in Lebanon, Uganda, Europe, the United States of America, and Australia [26, 31–34]. It is imperative to ensure an adequate budget, human resources, and salaries to increase the supply-side confidence of product marketing [23]. The possibility of mobilising local government funds and the inclusion of national and international donor organisations can be explored to increase the success of the vaccination campaign. Although Bangladesh has a literacy rate of 73.2% and 94% of households have a mobile phone, the use of the digital platform to register for vaccination service is still a challenge due to limited access to the Internet in rural and remote areas [35–37]. However, walk-in vaccination clinics have mitigated the difficulties of registration [38]. A breastfeeding corner can be established at vaccination clinics, or if not possible, lactating mothers should receive priority to get the vaccine in the queue. Grant Murewanhema et al. recommended the inclusion of COVID-19 vaccination in the antenatal-postnatal clinics and baby clinics to ensure safe and prompt vaccination of pregnant women and lactating women, respectively [39].

The main limitation of our study was that we did not conduct ethnographic observations of the vaccination programme in the target communities. However, we anticipate that the extensive interviews of both key informants and community members of varying backgrounds have reduced the information gap. A future study can be conducted, including ethnographic observations, and community mapping so that a more detailed view can be attained from the findings. The strength of our study is the inclusion of both the Government and Non-Government officials involved in the vaccination programme directly as key informants that have enriched the data quality from a programmatic perspective. Including both host and Rohingya community members for in-depth interviews enabled us to understand the community perceptions on a wider scale. Since the study was conducted to understand the vaccination programme at the Rohingya camp and host community of two sub-districts of Cox’s Bazar only, the study findings are not fully applicable to the vulnerable groups of other sites of Bangladesh but can provide an idea of the overall situation of the vaccination programmes of remote and rural parts of this country.

## Conclusion

Promotional activities are essential for improving the acceptance of vaccination in the community. The engagement of community leaders, providing transport facilities for vaccine recipients, especially for the elderly and pregnant women, having a breastfeeding corner at the vaccination site, having a proper contingency approach to tackle adverse effects of immunisation, adequate payment to the staff, and proper maintenance of the vaccine cold chain systems can have a positive impact on the vaccination programme. The findings from our study will help policymakers from the Government, the UN, and other humanitarian agencies to adapt and better address the issue of vaccine acceptance and strengthen the vaccination programme.

### Electronic supplementary material

Below is the link to the electronic supplementary material.

Supplementary Material 1

## Data Availability

The datasets used and/or analysed during the current study are available from the corresponding author on reasonable request.

## Abbreviations

COREQ: Consolidated criteria for reporting qualitative research
COVID-19: Coronavirus Disease 2019
IDI: In-depth Interview
IRB: Institutional Review Board
JRP: Joint Response Plan
KII: Key-Informant Interview
NGO: Non-Government Organisation
NSU: North South University
PLW: Pregnant and lactating women
PWD: People with disability
UH&FPO: Upazila Health & Family Planning Officer
WHO: World Health Organization

## References

[1] Centers for Disease Control and Prevention. Basics of COVID-19 [Available from: https://www.cdc.gov/coronavirus/2019-ncov/your-health/about-covid-19/basics-covid-19.html.

[2] Mayo Clinic. COVID-19 Timeline [Available from: https://www.mayoclinic.org/coronavirus-covid-19/history-disease-outbreaks-vaccine-timeline/covid-19.

[3] Al Jazeera. Bangladesh starts COVID vaccination drive use 2021 [Available from: https://www.aljazeera.com/news/2021/1/28/bangladesh-starts-covid-vaccination-drive.

[4] Anadolu Agency. Bangladesh starts nationwide COVID vaccination drive. 2021.

[5] I. S. C. G. (ISCG.). Joint Multi-Sector Needs Assessment - Rohingya Refugee May, 2021 [Available from: https://reliefweb.int/report/bangladesh/joint-multi-sector-needs-assessment-j-msna-bangladesh-rohingya-refugees-may-2021.

[6] G. of the P. R. of Bangladesh. Government of the People’s Republic of Bangladesh, Emergency MultiSector Rohingya Crisis Response Project (EMCRP) Environmental and Social Management Framework. January, 2019.

[7] ReliefWeb. COVID-19: Bangladesh Multi-Sectoral Anticipatory Impact and Needs Analysis - Needs Assessment Working Group, Bangladesh April 15 2020 [Available from: https://reliefweb.int/report/bangladesh/covid-19-bangladesh-multi-sectoral-anticipatory-impact-and-needs-analysis-needs.

[8] ReliefWeb. More than 100,000 Rohingya refugee children vaccinated against COVID in Bangladesh [EN/BN] 2022 [Available from: https://reliefweb.int/report/bangladesh/more-100000-rohingya-refugee-children-vaccinated-against-covid-bangladesh-enbn.

[9] USA for The UN Refugee Agency (UNHCR). Rohingya Refugee Crisis Explained 2022 [Available from: https://www.unrefugees.org/news/rohingya-refugee-crisis-explained/.

[10] Dhaka Tribune. Bangladesh’s Covid-19 vaccine stock to run out in one month. 2021.

[11] Reliefweb. COVID-19 vaccinations begin in Bangladesh’s Rohingya refugee camps 2021 [Available from: https://reliefweb.int/report/bangladesh/covid-19-vaccinations-begin-bangladesh-s-rohingya-refugee-camps.

[12] Bendau A, Plag J, Petzold MB, Ströhle A (2021). COVID-19 vaccine hesitancy and related fears and anxiety. Int Immunopharmacol.

[13] Soares P, Rocha JV, Moniz M, Gama A, Laires PA, Pedro AR (2021). Factors associated with COVID-19 vaccine hesitancy. Vaccines.

[14] Jalloh MF, Bennett SD, Alam D, Kouta P, Lourenço D, Alamgir M (2019). Rapid behavioral assessment of barriers and opportunities to improve vaccination coverage among displaced Rohingyas in Bangladesh, January 2018. Vaccine.

[15] Attwell K, Hannah A, Leask J (2022). COVID-19: talk of ‘vaccine hesitancy’lets governments off the hook. Nature.

[16] World Health Organization. COVID-19 Vaccination: WHO supports an effective campaign in Bangladesh while strengthening vaccine roll-out preparedness for Rohingya. 20 May 2021.

[17] Health Sector Cox’s Bazar. Emergency: Rohingya refugee/FDMN crisis in Cox’s Bazar District Bulletin #17. 2022.

[18] Alam AM. Providing COVID-19 vaccination to refugees and displaced people: lessons from the vaccine roll-out for the Rohingya refugees in Cox’s Bazaar, Bangladesh. Volume 10. The Lancet Regional Health-Southeast Asia; 2023. 10.1016/j.lansea.2022.100120 PMC9682057 36439026

[19] Sallam M (2021). COVID-19 vaccine hesitancy worldwide: a concise systematic review of vaccine acceptance rates. Vaccines.

[20] Tyagi K, Ghosh A, Nair D, Dutta K, Bhandari PS, Ansari IA et al. Breakthrough COVID19 infections after vaccinations in healthcare and other workers in a chronic care medical facility in New Delhi, India. Diabetes & Metabolic Syndrome: Clinical Research & Reviews. 2021;15(3):1007-8. 10.1016/j.dsx.2021.05.001 PMC8091733 33991805

[21] Lee CJ, Woo W, Kim AY, Yon DK, Lee SW, Koyanagi A (2022). Clinical manifestations of COVID-19 breakthrough infections: a systematic review and meta‐analysis. J Med Virol.

[22] Medicine Y. Comparing the COVID-19 Vaccines: How Are They Different? 2022 [Available from: https://www.yalemedicine.org/news/covid-19-vaccine-comparison.

[23] Evans WD, French J (2021). Demand creation for COVID-19 vaccination: overcoming vaccine hesitancy through social marketing. Vaccines.

[24] Riad A, Pokorná A, Attia S, Klugarová J, Koščík M, Klugar M (2021). Prevalence of COVID-19 vaccine side effects among healthcare workers in the Czech Republic. J Clin Med.

[25] Beatty AL, Peyser ND, Butcher XE, Cocohoba JM, Lin F, Olgin JE (2021). Analysis of COVID-19 vaccine type and adverse effects following vaccination. JAMA Netw open.

[26] Mahimbo A, Kang M, Sestakova L, Smith M, Dawson A (2022). Factors influencing refugees’ willingness to accept COVID-19 vaccines in Greater Sydney: a qualitative study. Aust N Z J Public Health.

[27] Chou W-YS, Budenz A (2020). Considering emotion in COVID-19 vaccine communication: addressing vaccine hesitancy and fostering vaccine confidence. Health Commun.

[28] Magee L, Knights F, Mckechnie DG, Al-Bedaery R, Razai MS (2022). Facilitators and barriers to COVID-19 vaccination uptake among ethnic minorities: a qualitative study in primary care. PLoS ONE.

[29] Rzymski P, Falfushynska H, Fal A (2022). Vaccination of ukrainian refugees: need for urgent action. Clin Infect Dis.

[30] Arnstein SR (1969). A ladder of citizen participation. J Am Inst Planners.

[31] Ali Z, Perera SM, Garbern SC, Diwan EA, Othman A, Ali J (2022). Variations in COVID-19 Vaccine Attitudes and Acceptance among Refugees and Lebanese Nationals Pre-and Post-Vaccine Rollout in Lebanon. Vaccines.

[32] Crawshaw AF, Farah Y, Deal A, Rustage K, Hayward SE, Carter J et al. Defining the determinants of vaccine uptake and undervaccination in migrant populations in Europe to improve routine and COVID-19 vaccine uptake: a systematic review. Lancet Infect Dis. 2022. 10.1016/S1473-3099(22)00066-4 PMC9007555 35429463

[33] Shaw J, Anderson KB, Fabi RE, Thompson CA, Harris M, Aljabbarin N (2022). COVID-19 vaccination intention and behavior in a large, diverse, US refugee population. Vaccine.

[34] Logie CH, Okumu M, Berry I, McAlpine A, Musoke DK, Hakiza R (2023). Multi-method findings on COVID-19 vaccine acceptability among urban refugee adolescents and youth in Kampala, Uganda. Glob Public Health.

[35] Government of the. People’s Republic of Bangladesh, Ministry of Health and Family Welfare. Health Bulletin 2019. June; 2020.

[36] National Institute of Population Research and Training (NIPORT) (2020). aI. Bangladesh Demographic and Health Survey 2017-18. Dhaka, Bangladesh, and Rockville.

[37] Molla MMA, Disha JA, Yeasmin M, Ghosh AK, Nafisa T (2021). Decreasing transmission and initiation of countrywide vaccination: key challenges for future management of COVID-19 pandemic in Bangladesh. Int J Health Plann Manag.

[38] Walk-. Covid-19 vaccination launched. The Business Standard; 2022.

[39] Murewanhema G, Dzinamarira T, Herrera H, Musuka G (2021). COVID-19 vaccination for pregnant women in Zimbabwe: a public health challenge that needs an urgent discourse. Public Health in Practice (Oxford England).

