# Supplementary material for: A formative research to explore the programmatic approach of vaccinating the Rohingya refugees and host communities against COVID-19 infection in Bangladesh
Source: BMC Health Serv Res. 2023 Aug 31;23:937. doi: 10.1186/s12913-023-09945-z (PMC10472616; doi:10.1186/s12913-023-09945-z)
Supplement: Supplementary file 1 — Supplementary Material 1 [file 12913_2023_9945_MOESM1_ESM.docx]

**Supplementary file – 1 Codebook**

| **Themes** | **Sub-themes** | **Codes** |
| --- | --- | --- |
| **Programmatic approach** | 1.1 Promotional activities: vaccine hesitancy survey and tailoring messages | - - 1. Date of the project start     2. Whether vaccine hesitancy survey was conducted     3. Vaccine promotional activities   1.1.3.1 Whether the promotional activities took place  1.1.3.2 Mode of promotional activities |
|  | 1.2 Inclusion of the community leaders in the decision-making process | 1.2.1 Whether the community members included in the decision-making process  1.2.2 Role of the community members in the decision-making process  1.2.2.1 Inclusion of pregnant women and elderlies |
|  | 1.3 Cold chain and quality of the vaccines | 1.3.1 Whether the project provided cold chain facility  1.3.2 Role of the project to ensure the quality of the vaccine  1.3.2.1 Transport facility  1.3.3 The process of maintaining cold chain and quality of the vaccines  1.3.4 Adequacy of the supply of the quality vaccines |
|  | 1.4 Preparatory mechanism to tackle unintended adverse effects of the vaccine | 1.4.1 Preparation to tackle the side-effects  1.4.1.1 Responsible person  1.3.1.2 Preparation of the healthcare facilities  1.4.2 Role of the project to tackle the side-effects |
| **Vaccination acceptance among the respondents** |  | 2.1 Hesitancy to take the vaccine  2.2 Cause of hesitancy  2.2.1 Death of people  2.2.2 Misinformation  2.2.3 Phobia  2.3 Reason of acceptance  2.3.1 Promotional activities  2.3.2 Acceptance among the doctors  2.4 Effect of post-vaccination side-effects  2.4.1 Side-effects  2.4.2 Severity of the side-effects  2.4.3 Management of the side-effects |
| **Barriers to the programme** | Barriers | 3.1.1 Community acceptance  3.1.2 Human resource  3.1.3 Logistics and equipment  3.1.4 Poor socio-economic condition  3.1.5 Communication  3.1.6 Registration process  3.1.7 Cultural context  3.1.8 Crowed control  3.1.9 Privacy for lactating mothers |
